# Appointment attendance at a remote rural dental training facility in Australia

**DOI:** 10.1186/1472-6831-13-36

**Published:** 2013-08-02

**Authors:** Ratilal Lalloo, Jenny M McDonald

**Affiliations:** 1 Rural, Remote & Indigenous Oral Health, School of Dentistry & Oral Health, Griffith Health Centre, Griffith University, Gold Coast, Australia; 2 Population & Social Health Research Programme, Griffith Health Institute, Griffith University, Gold Coast, Australia; 3 Griffith Health, Griffith University, Gold Coast, Australia; 4 DOH, Gold Coast campus, Griffith University, Gold Coast 4222, Australia

**Keywords:** Australia, Dental service, Education, Non-attended appointments, Remote, Rural

## Abstract

**Background:**

Non-attended appointments have impacts on the operations of dental clinics. These impacts vary from lost productivity, loss of income and loss of clinical teaching hours.

**Methods:**

Appointment data were analysed to assess the percentage of completed, failed to attend (FTA) and cancelled appointments at an Australian remote rural student dental clinic training facility. The demographic and time characteristics of FTA and cancelled appointments were analysed using simple and multivariate multinomial regression analysis, to inform interventions that may be necessary.

**Results:**

Over the 2-year study period a total of 3,042 appointments were made. The percentage of FTA was 21.3% (N = 648) and cancelled appointments 13.7% (N = 418). The odds of an FTA were in excess of 4 times higher in patients aged 19–25 years (OR = 4.1; 95% CI = 2.3-7.3) and 26–35 years (OR = 4.4; 95% CI = 2.5-7.9) compared to patients 65 years and older. The odds of an FTA was 2.3 (95% CI = 1.8-3.1) times higher in public patients compared to private patients. The odds of a cancellation was 1.7 (95% CI = 1.1-2.6) times higher on a Friday compared to a Monday and 1.8 (95% CI = 1.1-2.9) times higher on the last appointment of the day compared to the first appointment. For cancelled appointments, 71.3% were cancelled on the day of the appointment and 16.6% on the day before.

**Conclusions:**

Non-attended appointments (FTA or cancelled) were common at this remote rural dental clinic training facility. Efforts to reduce these need to be implemented; including telephonic reminders, educating the community on the importance of attending their appointments, block booking school children and double booking or arranging alternative activities for the students at times when non-attendance is common.

## Background

Non-attended appointments in a health care setting have significant productivity and economic impacts [1,2]. Productivity and budgetary targets are important aspects in the evaluation of a clinic’s performance, and patients’ not attending, either due to cancelled and failed to attend (FTA) appointments, may have significant consequences for the clinic in terms of sustainability and staffing. During times that patients do not attend the operations of the clinic continue and staff are paid, with no tangible productivity recorded. Opportunities are forgone to provide care to other patients if appointments are not attended. In a training facility, it has further impacts on the clinical experiences and operating hours of the students.

Levels of non-attended dental appointments range from 10-20%, but can vary from 5 to 50% [3-13]. Reasons for non-attendance most often relate to lack of transport, time and forgetfulness [8,13]. There are also suggestions that the level of non-attendance can differ by demographic characteristics such as gender, age, location (urban / rural), socioeconomic status, weather (season) and practice types [4,8,11,14-18]. Depression and moodiness, as well as anxiety and nervousness, have been suggested to predict cancelled or missed dental appointments [19,20]. For dental clinics, the oral health status of the person (higher caries experience and caries activity) may also predict appointment keeping behaviour [6,19]. The cultural appropriateness of the dental service might also impact on the level of non-attended appointments [21].

Interventions to reduce the level of non-attendance, including voice messages, text messages and paper reminders have been implemented, to varying degrees of success [3-5,8,10,14,22-25]. Rural clinical settings may experience higher levels of non-attendance than urban settings. This may be due to the travel distances for patients, lack and/or sharing of communication facilities such as telephones and mobiles and network coverage can occur in a remote setting, other pressing and urgent matters needing attention on the day. The literature is however currently too sparse to support this. Irrespective of the setting and reasons for non-attendance, it is important that clinics monitor and analyse non-attendance and make efforts to reduce the level of non-attendance of appointments.

The School of Dentistry and Oral Health at Griffith University started a remote rural clinical placement for final year dental students in February 2009 [26]. The dental training facility is located in Brewarrina, north-west New South Wales (NSW). Brewarrina is a remote rural, and predominantly Indigenous town, about 900 km from the Griffith University dental school, which is located in the City of the Gold Coast, south-east Queensland. The population of the town of Brewarrina is approximately 1,500; with 60 to 70% identifying themselves as Indigenous. The population of the wider Brewarrina Shire is a further 500 people, and this covers an area of ~20,000 square kilometres. The nearest large town is Dubbo, 374 km away, and the nearest other small towns are about 100 km distant. No dental services had been available in Brewarrina for many years prior to the commencement of this initiative in 2009. The clinic comprises 4 dental surgeries. In 2011 and 2012 students spent 3 and 4-weeks respectively at this rural clinic and are generally rotated in groups of 8. Student accommodation and travel are provided: the latter being a 12 hour road journey. Staffing at the clinic includes a supervising dentist, a dental assistant and a receptionist. The clinic provides health promotion, prevention and basic dental services to the community of the town and surrounding areas. The students experience a setting that would be unique for most, and this placement we hope will make them appreciate the general and oral health burden experienced by rural, remote & Indigenous communities as well as the risk factors they confront on a daily basis [26].

The objective of the study is to provide an overview of the appointment patient flow at the Brewarrina Dental Clinic in 2011 and 2012; and to assess the patient demographic and time characteristics associated with completed, failed to attend (FTA) and cancelled appointments.

## Methods

At the university dental clinic in the Gold Coast, all patient records, including the appointment system, are captured electronically, using the Titanium patient management system of Spark Dental Technology (https://www.spark-dental.com/solutions/titanium/). The same system was installed in the rural dental clinic in Brewarrina, and is linked to the main server on the Gold Coast.

The demographic and time data attached to each appointment for 2011 and 2012 were extracted from the system and analysed to investigate and compare the characteristics of the various appointments, i.e. completed, FTA and cancelled. The percentage of completed, FTA and cancelled appointments were analysed in terms of gender, age, residential location, type of patient (public and private), day of the week, time of day, a combination of time of day and day of the week, and by month. Appointment keeping patterns by the number of appointments made per patient were also explored. The data extracted were not captured specifically for this study, but routinely collected as per clinical operations.

In Australia dental care services are provided by both the private and public sectors. However more than 80% of dentists work in the private sector located largely in major cities. To be eligible for dental care in the public service eligibility criteria need to be met. For adults to be eligible they must be in receipt of benefits from either a Pensioner Concession or Seniors Health Card (http://www.health.qld.gov.au/oralhealth/services/adult.asp). All resident children four years of age or older who have not completed Year 10 of secondary school are eligible (http://www.health.qld.gov.au/oralhealth/services/school.asp). Children less than 4 year of age who have a health care card or are dependents of health care card holders are also eligible for dental care in the public service.

The data utilised in this analysis are routinely collected and therefore ethics approval was not required. This is based on the standards and principles of the Australian National Statement on Ethical Conduct of Human Research and further confirmed by Griffith University Human Research Ethics Committee. No patient names have been disclosed in the manuscript. All patients sign an informed consent when they attend the clinic, and the following statement is included in the consent form: *“I also give consent to Griffith University to use my information for clinical, education, research and development purposes, so long as my name is not disclosed in any reports or published documents.”*

The data analysis included assessing the frequency distribution of completed, FTA and cancelled appointments, by patient demographic and time characteristics. The associations between the demographic and time characteristics of appointments and their status (completed, FTA or cancelled) were assessed by conducting a simple and multivariate multinomial regression analysis. Multinomial logistic regression is used to model nominal outcome variables. For the regression analysis the odds ratio (OR) and 95% confidence interval (CI) are presented for FTA and cancelled appointments, compared to completed appointments, for the demographic and time characteristic variables. Odds ratios with a 95% confidence interval excluding 1 were considered as statistically significant, and are indicated in bold font in the results table.

## Results

In 2011 there were 8 student rotations of 3-weeks each across the academic year. A total of 1,862 appointments were made by 888 patients. In 2012 there were 6 student rotations of 4-weeks each across the academic year. A total of 1,180 appointments were made by 674 patients. Of all the appointments more than half were for people living in Brewarrina (N = 1,576; 53.3%). Of the people from outside Brewarrina, 45.5% (N = 628) were from Bourke (almost 100 km away); 16.1% (N = 223) from Lightning Ridge (210 km away) and the balance from a number of surrounding towns in the region.

53.0% (N = 1611) of appointments were for females. Excluding the unknown ages (N = 71; 2.3%), a quarter (N = 792) of all appointments were for children (< 19 years of age). Of the adults most were aged 36–49 years (N = 649). In 9.4% (N = 287) of records the type of patient (private or public) was not recorded. These may be patients who made an appointment but who then never visited the clinic (missed or cancelled the appointment), so the only detail recorded was when the appointment was made. Of the patients lacking this information, 63.0% did not attend any of their appointments. Of those where the information was recorded, almost three-quarters were public patients. The balance were private patients, who paid a fee for the care provided.

**Figure 1:**
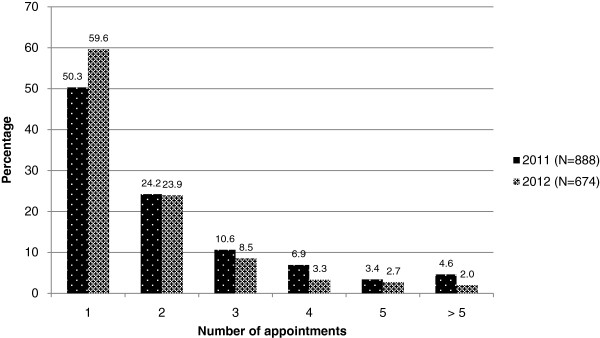
Percentage distribution of the number of appointments per patient.

A total of 888 patients made appointments in 2011, 50.3% (N = 447) made a single appointment and 24.2% (N = 215) made two appointments (Figure 1). Of those who made a single appointment, 30.9% did not attend it. Of these 58.0% were FTAs and 42.0% cancellations. Of the 215 patients who made two appointments, 44.2% attended one and 21.4% missed both. A total of 674 patients made appointments in 2012, 59.6% (N = 402) made a single appointment and 23.9% (N = 161) made two appointments. Of those who made a single appointment, 25.0% did not attend it, with 86.0% of these being an FTA. Of the 161 patients who made two appointments, 34.2% attended one and 14.3% missed both.

In 2011 and 2012 a total of 3,042 appointments were made. The combined percentage of non-attendance (FTAs and cancellations) was 35.0% (N = 1,066), with the percentage of FTAs at 21.3% (N = 648) and cancelled appointments at 13.7% (N = 418). Table 1 shows the number and percentage of completed, FTAs and cancelled appointments combined across both years of the study by a number of demographic and time-related characteristics and the simple (unadjusted) multinomial regression analysis (OR and 95% CI). The unadjusted odds of an FTA were higher across all age groups (in particular those aged 19–35 years) compared to the elderly 65 years and older; in public patients compared to private patients; in patients from the local town compared to other towns; and in almost all months of clinical operation compared to February. The odds of a cancellation were higher on a Friday compared to a Monday. The odds of both an FTA and cancellation were higher in females compared to males and in patients with no contactable telephone number recorded compared to those with one.

**Table 1:** **Distribution of demographic and time characteristics and simple (unadjusted) odds ratios (OR) for appointment status (N = 3,042)**^**a**^

**Variable**	**Completed**	**FTA**^**b**^	**FTA**	**Cancelled**	**Cancelled**
	**N**^**c **^**(%)**	**N (%)**	**OR**^**d**^		**OR**
			**(95% CI)**^**e**^	**N (%)**	**(95% CI)**
Overall	1976 (65.0)	648 (21.3)	-	418 (13.7)	-
*Residential suburb*					
Brewarrina	991 (62.9)	398 (25.3)	**2.2 (1.7-2.9)**	187 (11.9)	0.9 (0.7-1.2)
Bourke	426 (67.8)	122 (19.4)	**1.6 (1.1-2.2)**	80 (12.7)	0.9 (0.6-1.3)
Lightning Ridge	158 (70.9)	27 (12.1)	0.9 (0.6-1.5)	38 (17.0)	1.2 (0.8-1.8)
Other*	381 (71.9)	69 (13.0)	1	80 (15.1)	1
*Gender*					
Female	998 (61.9)	362 (22.5)	**1.3 (1.1-1.5)**	251 (15.6)	**1.5 (1.2-1.8)**
Male*	971 (68.6)	279 (19.7)	1	165 (11.7)	1
*Age group*					
< 19	535 (67.6)	184 (23.2)	**4.1 (2.4-7.0)**	73 (9.2)	1.0 (0.6-1.6)
19-25	225 (60.8)	100 (27.0)	**5.3 (3.0-9.3)**	45 (12.2)	1.4 (0.9-2.4)
26-35	257 (59.7)	119 (27.5)	**5.5 (3.2-9.6)**	56 (13.0)	1.5 (0.9-2.5)
36-49	405 (62.4)	125 (19.3)	**3.7 (2.1-6.4)**	119 (18.3)	**2.1 (1.3-3.3)**
50-64	342 (69.2)	80 (16.2)	**2.8 (1.6-4.9)**	72 (14.6)	1. 5 (0.9-2.4)
65 + *	191 (81.6)	16 (6.8)	1	27 (11.5)	1
*Type of patient*					
Public	1322 (65.2)	446 (22.0)	**2.1 (1.6-2.7)**	259 (12.8)	1.2 (0.9-1.6)
Private*	551 (75.7)	89 (12.2)	1	88 (12.1)	1
*Contact telephone*					
*number*					
Yes*	1752 (67.6)	491 (19.0)	1	348 (13.4)	1
No	224 (49.7)	157 (34.8)	**2.5 (2.0-3.1)**	70 (15.5)	**1.6 (1.2-2.1)**
*Day of week*					
Monday*	377 (64.4)	144 (24.6)	1	64 (10.9)	1
Tuesday	420 (65.4)	124 (19.3)	0.8 (0.6-1.0)	98 (15.3)	1.4 (1.0-1.9)
Wednesday	492 (65.5)	159 (21.2)	0.9 (0.7-1.1)	100 (13.3)	1.2 (0.8-1.7)
Thursday	440 (66.8)	142 (21.5)	0.9 (0.7-1.1)	77 (11.7)	1.0 (0.7-1.5)
Friday	247 (61.0)	79 (19.5)	0.8 (0.6-1.2)	79 (19.5)	**1.9 (1.3-2.7)**
*Time of day*					
1^st^ appointment*	318 (65.8)	103 (21.3)	1	62 (12.8)	1
Morning to lunch	1011 (66.3)	286 (18.7)	0.9 (0.7-1.1)	229 (15.0)	1.2 (0.9-1.6)
After Lunch	484 (64.5)	184 (24.5)	1.2 (0.9-1.6)	82 (10.9)	0.9 (0.6-1.2)
Last appointment	163 (58.4)	75 (26.9)	1.4 (1.0-2.0)	41 (14.7)	1.3 (0.8-2.0)
*Month*					
February*	82 (70.7)	13 (11.2)	1	21 (18.1)	1
March	406 (62.6)	131 (20.2)	**2.0 (1.1-3.8)**	112 (17.3)	1.1 (0.6-1.8)
April	205 (55.9)	105 (28.6)	**3.2 (1.7-6.1)**	57 (15.5)	1.1 (0.6-1.9)
May	287 (66.3)	96 (22.2)	**2.1 (1.1-4.0)**	50 (11.5)	0.7 (0.4-1.2)
June	135 (69.2)	50 (25.6)	**2.3 (1.2-4.6)**	10 (5.1)	**0.3 (0.1-0.7)**
July	189 (72.4)	61 (23.4)	**2.0 (1.1-3.9)**	11 (4.2)	**0.2 (0.1-0.5)**
August	374 (63.2)	104 (17.6)	1.8 (0.9-3.3)	114 (19.3)	1.2 (0.7-2.0)
September	298 (69.5)	88 (20.5)	1.9 (1.0-3.5)	43 (10.0)	0.6 (0.3-1.0)

* Reference category. ^a^Sample sizes are smaller for some variables due to missing data. ^b^Sample. ^c^Failed to attend. ^d^Odds ratio. ^e^95% confidence interval.

Table 2 shows the frequency distribution and multivariate (fully adjusted) multinomial regression analysis. The multivariate analysis shows that the odds of an FTA were 2.5 (95% CI = 1.8-3.6) times higher for people living in Brewarrina compared to people from distant towns. The odds of a cancellation were 1.5 (95% CI = 1.2-2.0) times higher in females compared to males. The odds of an FTA was in excess of 4 times higher in patients aged 19–25 years (OR = 4.1; 95% CI = 2.3-7.3) and 26–35 years (OR = 4.4; 95% CI = 2.5-7.9) compared to patients 65 years and older. The odds of an FTA or cancellation were 2.3 (95% CI = 1.8-3.1) and 1.5 (95% CI = 1.1-2.0) times higher respectively in public patients compared to private patients. The odds of an FTA were 1.8 times (95% CI = 1.3-2.4) higher amongst patients for whom a contact telephone/mobile number was not recorded on the system compared to those with a contact recorded. The odds of a cancellation were 1.7 times (95% CI = 1.1-2.6) higher on a Friday compared to a Monday and 1.8 (95% CI = 1.1-2.9) times higher on the last appointment of the day compared to the first appointment. Compared to February, the odds of an FTA were higher during all other months of the clinic’s operation.

**Table 2:** Distribution of demographic and time characteristic and multivariate (fully-adjusted) odds ratios (OR) for appointment status (N = 2,692)

**Variable**	**Completed N**^**a **^**(%)**	**FTA**^**b **^**N (%)**	**FTA OR**^**c **^**(95% CI)**^**d**^	**Cancelled N (%)**	**Cancelled OR (95% CI)**
Overall	1851 (68.8)	511 (19.0)	-	330 (12.3)	-
*Residential suburb*					
Brewarrina	936 (65.7)	337 (23.6)	**2.5 (1.8-3.6)**	152 (10.7)	0.8 (0.6-1.2)
Bourke	408 (69.6)	105 (17.9)	**2.0 (1.3-2.9)**	73 (12.5)	0.9 (0.7-1.4)
Lightning Ridge	155 (73.1)	22 (10.4)	1.2 (0.7-2.0)	35 (16.5)	1.1 (0.7-1.7)
Other*	352 (75.1)	47 (10.0)	1	70 (14.9)	1
*Gender*					
Female	931 (65.5)	287 (20.2)	1.2 (1.0-1.5)	204 (14.3)	**1.5 (1.2-2.0)**
Male*	920 (72.4)	224 (17.6)	1	126 (9.9)	1
*Age group*					
< 19	484 (71.3)	137 (20.2)	**2.3 (1.3-4.1)**	58 (8.5)	0.6 (0.4-1.1)
19-25	214 (64.5)	85 (25.6)	**4.1 (2.3-7.3)**	33 (9.9)	1.0 (0.6-1.9)
26-35	241 (62.1)	98 (25.3)	**4.4 (2.5-7.9)**	49 (12.6)	1.3 (0.7-2.2)
36-49	388 (64.8)	105 (17.5)	**3.1 (1.8-5.5)**	106 (17.7)	**1.9 (1.1-3.1)**
50-64	334 (71.8)	70 (15.1)	**2.9 (1.6-5.2)**	61 (13.1)	1. 4 (0.8-2.4)
65 + *	190 (83.0)	16 (7.0)	1	23 (10.0)	1
*Type of patient*					
Public	1303 (66.1)	423 (21.5)	**2.3 (1.8-3.1)**	244 (12.4)	**1.5 (1.1-2.0)**
Private*	548 (75.9)	88 (12.2)	1	86 (11.9)	1
*Contact telephone*					
*number*					
Yes*	1647 (70.1)	408 (17.4)	1	293 (12.5)	1
No	204 (59.3)	103 (29.9)	**1.8 (1.3-2.4)**	37 (10.8)	1.1 (0.7-1.6)
*Day of week*					
Monday*	359 (68.4)	114 (21.7)	1	52 (9.9)	1
Tuesday	403 (70.7)	93 (16.3)	0.8 (0.6-1.2)	74 (13.0)	1.1 (0.7-1.6)
Wednesday	449 (68.2)	129 (19.6)	1.0 (0.7-1.3)	80 (12.2)	1.0 (0.7-1.5)
Thursday	410 (71.4)	108 (18.8)	0.9 (0.6-1.2)	56 (9.8)	0.8 (0.5-1.2)
Friday	230 (63.0)	67 (18.4)	1.3 (0.9-1.9)	68 (18.6)	**1.7 (1.1-2.6)**
*Time of day*					
1^st^ appointment*	298 (69.3)	80 (18.6)	1	52 (12.1)	1
Morning to lunch	951 (69.6)	231 (16.9)	0.9 (0.7-1.3)	185 (13.5)	1.1 (0.8-1.5)
After Lunch	455 (69.6)	139 (21.3)	1.2 (0.8-1.6)	60 (9.2)	0.9 (0.6-1.3)
Last appointment	147 (61.0)	61 (25.3)	1.3 (0.9-1.9)	33 (13.7)	**1.8 (1.1-2.9)**
*Month*					
February*	78 (73.6)	9 (8.5)	1	19 (17.9)	1
March	346 (68.8)	89 (17.7)	**2.3 (1.1-4.7)**	68 (13.5)	0.9 (0.5-1.6)
April	194 (61.2)	81 (25.6)	**4.7 (2.2-10.0)**	42 (13.2)	0.9 (0.5-1.8)
May	275 (68.4)	84 (20.9)	**3.3 (1.6-7.0)**	43 (10.7)	0.7 (0.4-1.3)
June	132 (71.7)	43 (23.4)	**3.5 (1.6-7.8)**	9 (4.9)	**0.3 (0.1-0.8)**
July	180 (74.4)	54 (22.3)	**3.6 (1.7-8.0)**	8 (3.3)	**0.2 (0.0-0.5)**
August	361 (65.5)	85 (15.4)	**2.5 (1.2-5.2)**	105 (19.1)	1.3 (0.7-2.2)
September	285 (73.6)	66 (17.1)	**2.9 (1.3-6.1)**	36 (9.3)	0.6 (0.3-1.1)

* Reference category. ^a^Sample. ^b^Failed to attend. ^c^Odds ratio. ^d^95% confidence interval.

A more detailed analysis of the day of the week and time of day showed that about a third of the last appointments on a Monday (N = 19; 31.1%) and Thursday (N = 21; 31.3%) were an FTA. The first appointment on a Monday (N = 21; 28.4%) and post lunch on a Thursday (N = 49; 28.0%) were also common for FTAs.

For cancelled appointments in 2011, 71.3% were cancelled on the day of the appointment and 16.6% on the day before. While cancelled appointments were fewer in 2012, 60% were cancelled on the day of the appointment and 24% the day before.

## Discussion

The findings that Brewarrina residents were at higher odds to FTA are not surprising because people living nearby are aware that the clinic is easily accessible at another time. Females were at higher odds to cancel and this may be due to family (care of children) needs taking priority [21]. Young adults were at more than 4 times higher odds to FTA compared to older patients and this may be due to forgetfulness, work or other issues taking priority such as the care of young children. Public patients were at higher odds to FTA and this may be due to public patients (generally of lower socioeconomic status) not having access to a telephone to cancel appointments, family obligations and other urgent priorities needing their attention at short notice. The percentage of public patients with a recorded contact telephone on the system was 82.6% compared to 95.6% amongst private patients. The lack of a telephone number on the system may be due to the patient not having one or because it was not recorded.

It appears that some days of week and times of the day are at higher odds of FTAs and cancellations. These appointments may need to be considered for double bookings or only for patients who have a contact number and confirming the appointments a working day before and / or on the day itself. Activities away from the clinic, such as school visits, may need to be considered for a Monday morning.

Almost 90% of appointments were cancelled on the day or the day before the appointment. Late cancellations cannot be filled as alternative patients for these appointment slots are not easily found in a remote rural setting.

The high percentage of non-attended appointments may also be due to the structure and operations of the clinic, which is largely based on the usual public sector system of specific appointments [21]. This rigidity may not be appropriate for rural Indigenous settings due to differences in the concept of time. However flexibility in appointment arrangements needs to be balanced with the optimal operation of the clinic.

The current patient management system does not allow easy retrieval of the types of appointments patients have made. The type of appointment relates to whether it is an emergency or a non-emergency, and more specifically appointments for preventive, periodontic, oral surgery or restorative care. It was therefore not possible to investigate missed appointments by types of care. Data on the oral health status of patients was not easily retrievable and therefore the relationship between this and appointment keeping was not possible. None of variables included in the analysis had more than 10% missing data; and was only high for the payor type (public or private) variable, at 9.4% (N = 287). Most other variables either had no missing data or less than 3%; for example for residential suburb it was 2.8% (N = 85), age 2.3% (N = 71) and gender 0.5% (N = 16). This percentage of missing data in a sample of more than 3,000 appointments is unlikely to significantly impact the findings of the study. The relationships between appointment status and the demographic and time variables essentially remained unchanged regarding statistical significance between the simple and multivariate regression analyses.

Non-attended appointments (FTA or cancelled) were common at this rural dental clinic. Whilst there are a significant number of remote and rural dental clinical projects in Australia [27-32], a detailed analysis of appointment keeping in these settings is not common. A study in rural South Australia showed that the prevalence of missed appointments was lower than our study at about 13.5% and most were due to failure to attend [21]. The setting was however larger than Brewarrina, with a population of almost 14,000 people. The literature on appointment keeping generally report a non-attendance range from 10-20% [6,7,9,10,12,16], however at our remote rural clinic the percentage of non-attendance is much higher at 35%. This is similar to a study in rural India where almost a third of patients missed their recall appointment [8].

Efforts to reduce missed appointments need to be implemented, including telephonic reminders a day before and / or on the day [3,5,10,25], educating the community on the importance of attending their appointments [1], block booking school children with reminders to the school and double booking on the times when non-attendance is common. At the point of making an appointment a recording of a contactable telephone and / or mobile number is important to allow for appointment reminders to be sent. In this rural setting it would be inappropriate (and unethical) to be punitive to patients who FTA and cancel appointments as the local and surrounding communities do not have alternative easily accessible dental services in the area, and the extenuating circumstances to missing appointments needs to be fully understood prior to considering a harsher policy related to missed appointments.

## Conclusion

The findings show that the percentage of non-attended appointments at this remote rural dental clinic was high at 35%, and that proximity to the clinic, gender, age, type of patient (public or private), time of day and day of week were significantly related to non-attendance of booked appointments. Residents from the local community were at higher odds to FTA compared to people from distant towns. Adults aged 19–35 years were at higher odds to FTA compared to the elderly. Females were at higher odds to cancel their appointments compared to males.

## Competing interests

The authors declare that they have no competing interests.

## Authors’ contributions

RL conceptualised, designed, analysed and drafted the manuscript. JM provided the data and provided editorial support. All authors read and approved the final manuscript.

## Pre-publication history

The pre-publication history for this paper can be accessed here:

http://www.biomedcentral.com/1472-6831/13/36/prepub
